# Reply to Gurney, T.; Ronca, F. Comment on “Hack et al. Effect of Guarana (*Paullinia cupana*) on Cognitive Performance: A Systematic Review and Meta-Analysis. *Nutrients* 2023, *15*, 434”

**DOI:** 10.3390/nu15082001

**Published:** 2023-04-21

**Authors:** Brian Hack, Eduardo Macedo Penna, Tyler Talik, Rohan Chandrashekhar, Mindy Millard-Stafford

**Affiliations:** 1 School of Biological Sciences, Georgia Institute of Technology, Atlanta, GA 30318, USA; bhack@gatech.edu (B.H.); ttalik3@gatech.edu (T.T.); rohanchandra@gatech.edu (R.C.); 2 Physical Education Faculty, Federal University of Pará, Castanhal 68746-630, PA, Brazil; eduardomp@ufpa.br

We thank Dr. Gurney for his interest and comment [1] on our recent systematic review and meta-analysis quantifying the effect of Guarana on cognitive performance [2]. Our paper compared the acute effect of Guarana ingestion versus a placebo on a variety of cognitive tasks without the additional influence of exercise. Our findings indicated that Guarana did not provide a significant effect on cognitive performance when aggregating effect sizes (ES) across all tasks and outcome variables reported (e.g., accuracy and response time) within each individual study. However, a sub-group analysis revealed a difference between accuracy and response time measures across the included studies; specifically, response time was significantly faster with Guarana compared to a control trial, although the effect size (ES = 0.202) was considered “small”.

We are pleased that Dr. Gurney and colleagues found similar results in their recent study [3], observing a faster response in choice reaction time with Guarana (unrelated to exercise). Unfortunately, the date of our systematic review preceded the publication of this study, and therefore it was not included in our meta-analysis. Notably, if we add this single test to our sub-group analysis (with data provided in the published paper), the ES for response time with Guarana, although significant, remains “small” (ES = 0.225). In summary, we concur with the comment [1] that additional research is warranted to understand if Guarana provides additional advantages in cognitive performance compared to caffeine alone while under either resting or exercise settings.

## References

[1] Gurney T., Ronca F. (2023). Comment on Hack et al. Effect of Guarana (*Paullinia cupana*) on Cognitive Performance: A Systematic Review and Meta-Analysis. *Nutrients* 2023, *15*, 434. Nutrients.

[2] Hack B., Penna E.M., Talik T., Chandrashekhar R., Millard-Stafford M. (2023). Effect of Guarana (*Paullinia cupana*) on Cognitive Performance: A Systematic Review and Meta-Analysis. Nutrients.

[3] Gurney T., Bradley N., Izquierdo D., Ronca F. (2022). Cognitive Effects of Guarana Supplementation with Maximal Intensity Cycling. Br. J. Nutr..

